# Characterization of the *Theileria parva* sporozoite proteome

**DOI:** 10.1016/j.ijpara.2017.09.007

**Published:** 2018-03

**Authors:** James Nyagwange, Edwin Tijhaar, Nicola Ternette, Fredrick Mobegi, Kyle Tretina, Joana C. Silva, Roger Pelle, Vishvanath Nene

**Affiliations:** a International Livestock Research Institute, P.O. Box 30709, Nairobi, Kenya; b Cell Biology and Immunology Group, Wageningen University, The Netherlands; c The Jenner Institute, Nuffield Department of Medicine, University of Oxford, UK; d Institute for Genome Sciences, University of Maryland School of Medicine, Baltimore, MD, USA; e Department of Microbiology and Immunology, University of Maryland School of Medicine, Baltimore, MD, USA; f Department of Infection and Immunity, South Australian Health and Medical Research Institute, North Terrace, Adelaide 5000, South Australia, Australia

**Keywords:** *Theileria*, Sporozoites, Proteomics, MudPIT, Antigens, East Coast fever

## Abstract

East Coast fever is a lymphoproliferative disease caused by the tick-borne protozoan parasite *Theileria parva*. The sporozoite stage of this parasite, harboured and released from the salivary glands of the tick *Rhipicephalus appendiculatus* during feeding, invades and establishes infection in bovine lymphocytes. Blocking this initial stage of invasion presents a promising vaccine strategy for control of East Coast fever and can in part be achieved by targeting the major sporozoite surface protein p67. To support research on the biology of *T. parva* and the identification of additional candidate vaccine antigens, we report on the sporozoite proteome as defined by LC–MS/MS analysis. In total, 4780 proteins were identified in an enriched preparation of sporozoites. Of these, 2007 were identified as *T. parva* proteins, representing close to 50% of the total predicted parasite proteome. The remaining 2773 proteins were derived from the tick vector. The identified sporozoite proteins include a set of known *T. parva* antigens targeted by antibodies and cytotoxic T cells from cattle that are immune to East Coast fever. We also identified proteins predicted to be orthologs of *Plasmodium falciparum* sporozoite surface molecules and invasion organelle proteins, and proteins that may contribute to the phenomenon of bovine lymphocyte transformation. Overall, these data establish a protein expression profile of *T. parva* sporozoites as an important starting point for further study of a parasitic species which has considerable agricultural impact.

## Highlights

• 2007 *Theileria parva* proteins expressed in the sporozoite were identified.
• Proteins include known *T. parva* antigens targeted by antibodies and cytotoxic T cells.
• Proteins predicted to be orthologs of *Plasmodium falciparum* sporozoite surface molecules were identified.
• Proteins predicted to be orthologs of *P. falciparum* invasion organelle proteins were identified.
• Proteins that may contribute to the phenomenon of bovine lymphocyte transformation were identified.

## Graphical abstract

**Figure:**
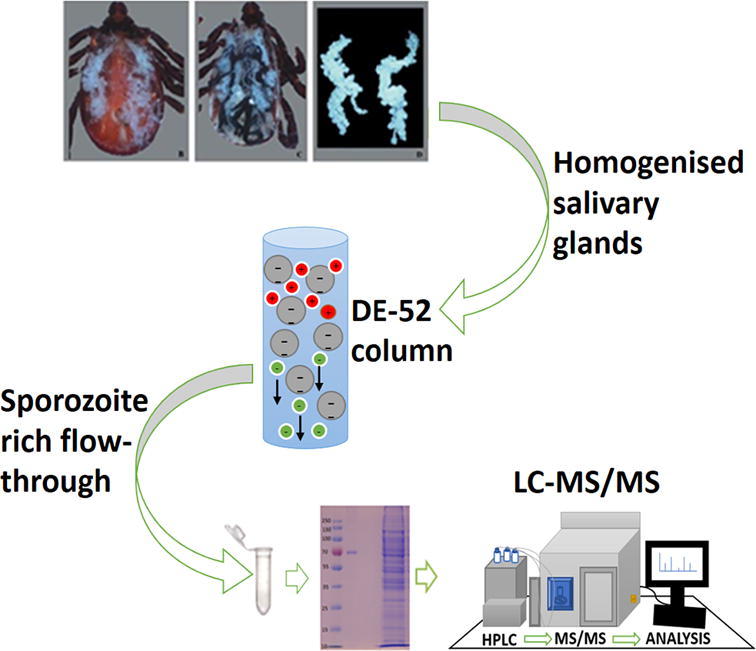

## 1 Introduction

East Coast fever (ECF), a fatal bovine disease, is caused by the apicomplexan parasite *Theileria parva* (Norval et al., 1992)*.* The disease kills approximately one million cattle every year in Africa and causes significant economic losses to small-hold farmers in eastern, central and southern Africa of approximately $300 million annually (reviewed in Nene et al., 2016). These estimates were derived several years ago and it is highly likely that the current losses due to ECF are much larger. Vaccination against ECF by an infection and treatment method (ITM) is available (Radley et al., 1975). For control of ECF in southern Africa, except South Africa where vaccination is not permitted as it is free of ECF, ITM is performed with a single parasite isolate, and in parts of eastern Africa a composite ITM vaccine, the Muguga cocktail, is used (Morzaria et al., 2000). Although ITM confers solid and long-lived immunity it has disadvantages in that it is an unstable and potentially lethal product, and animals vaccinated by the ITM protocol remain life-long asymptomatic carriers of the parasite that pose risks for spread of the disease. It also requires a liquid nitrogen cold chain for storage and oxytetracycline for co-treatment (reviewed in Di Giulio et al., 2009). The vaccine is expensive and laborious to produce, and it requires skilled personnel for delivery (reviewed in Di Giulio et al., 2009). Therefore, development of a subunit vaccine that is easier to produce and with minimal risks is necessary.

Although classified in the phylum Apicomplexa, aspects of the biology of *Theileria* are atypical of such organisms. For example, *Theileria* sporozoites are not motile, they have a less defined apical complex and host cell entry is not orientation-specific (Shaw, 2003). Sporozoites bind and enter bovine lymphocytes by a ‘‘zippering’’ process of the host and the sporozoite cell membranes (Fawcett et al., 1982). After entry into the host cell, rhoptries/microspheres are discharged with a rapid escape of the sporozoite from the surrounding host cell membrane. Sporozoites differentiate to the schizont stage, which resides in the host cytoplasm surrounded by host cell microtubules that are seemingly nucleated by parasite molecules (reviewed in Shaw, 2003). Schizont-infected cells acquire a cancer-like phenotype and are the cause of disease (Norval et al., 1992). Sporozoites also bind and enter macrophages/afferent lymph veiled cells by a zippering process, but these cells are not susceptible to transformation and sporozoites appear to only differentiate to an early schizont stage (Shaw et al., 1993). Several host cell signalling pathways that contribute to host cell transformation have been studied (Dobbelaere and Küenzi, 2004, Dessauge et al., 2005). In addition, parasite molecules that are associated with the transformation process have been tentatively identified by taking advantage of comparative genomics and cancer biology (Shiels et al., 2006, Hayashida et al., 2012, Tretina et al., 2015). A parasite-encoded prolyl isomerase has been recently identified as playing a key role in this complex host cell transformation process (Marsolier et al., 2015).

There is good evidence for roles of *T. parva* sporozoite and schizont antigens as candidate vaccine antigens. Several schizont antigens are targets of cytotoxic T lymphocytes (CTLs) that lyse schizont-infected cells (Graham et al., 2006, Graham et al., 2007, Graham et al., 2008) and two sporozoite antigens are targets of neutralising antibodies (Dobbelaere et al., 1985, Shapiro et al., 1987). One of the latter antigens, p67, has been shown to consistently protect a proportion of immunised cattle against challenge with needle-injected sporozoites and from infected ticks in field trials (reviewed in Nene et al., 2016). The p67-based vaccine might be improved by including additional antigens that can neutralise sporozoite infectivity.

As a first step towards improving our molecular understanding of the biology of *T. parva* we have undertaken a Multidimensional Protein Identification Technology (MudPIT) LC–MS/MS approach (reviewed in Schirmer et al., 2003) to characterise the proteome of *T. parva* sporozoites purified by ion-exchange chromatography (Musoke et al., 1992). We have employed bioinformatic tools to analyse 2007 parasite proteins that we were able to detect and identified *T. parva* orthologs of *Plasmodium falciparum* invasion organelle proteins, calcium signalling proteins and surface proteins. Some of these may represent novel *T. parva* vaccine candidates.

## 2 Materials and methods

### 2.1 Animals and sporozoite production

The procedure for sporozoites production has been previously described in detail (Patel et al., 2016). Briefly, cattle aged 7–9 months, previously managed under a strict acaricide regime, were maintained for at least 2 months and tested for exposure to *T. parva*, *Theileria mutans*, *Babesia bigemina* and *Anaplasma marginale* using ELISA and, in certain instances, with PCR analysis (Morzaria et al., 1999, Odongo et al., 2010). The naïve cattle were then inoculated with *T. parva* stabilates of the Muguga isolate and infection of the cattle was assessed. A colony of *Rhipicephalus appendiculatus* nymphs, previously tested for viruses such as Bovine Viral Diarrhea virus (BVDV) and Bunya viruses, and demonstrated to be free from tick-borne haemoparasites (Patel et al., 2016), was then applied to the infected cattle. The fully-fed nymphs were collected and incubated for 6 weeks at 24 °C to induce moulting into adults and infection rates assessed by microscopy as described before (Büscher and Tangus, 1986). A total of 300 adult ticks infected with *T. parva* Muguga isolate (mean infection rate of 167 acini per tick) were allowed to feed on rabbits to induce sporozoite maturation. After 4 days, the tick salivary glands were dissected from the ticks. All animal procedures described in this article were approved by International Livestock Research Institute’s (ILRI, (Kenya)), Institute Animal Care and Use Committee (IACUC File Number 2014.01).

### 2.2 Sporozoite purification

Dissected tick salivary glands were collected in a tube with 2 ml of cold PBS, transferred to a chilled glass hand homogenizer (‘UNI-FORM’ (England), 4–5 ml of homogenizer) and disrupted by gentle up, down and circular motion of the pestle until a uniform cloudy suspension was formed. The suspension was then centrifuged at 1000*g* at 4 °C for 5 min. The resulting supernatant containing sporozoites was applied to an 8 ml diethylaminoethyl cellulose (DE-52; Sigma–Aldrich (USA) Cat. No. D3764) column (Musoke et al., 1992). Flow-through fractions of 3.5 ml were collected in siliconised 10 ml conical bottomed glass tubes on ice. The sporozoite-rich cloudy fractions (numbers 2–5) were pooled and centrifuged at 12,000*g* at 4 °C for 5 min. The resulting sporozoite-rich small yellowish pellet was resuspended in SDS–PAGE sample buffer and heated at 100 °C for 5 min prior to fractionation on 10% one-dimensional SDS–PAGE and staining with Coomassie Blue.

### 2.3 In-gel trypsin digestion for LC–MS/MS analysis

The gel lane containing sporozoite material was excised and divided into four fractions of different size proteins (>100 kDa, 100–55 kDa, 55–35 kDa and <35 kDa). The gel pieces were de-stained overnight in 50% methanol, 5% acetic acid in water. After dehydration with acetonitrile, proteins were reduced with 10 mM DTT in 100 mM ammonium bicarbonate, and subsequently alkylated using 50 mM iodoacetamide in ammonium bicarbonate for 30 min at room temperature. Following washing of the gel pieces in 100 mM ammonium bicarbonate, proteolytic digestion was carried out with 100 ng of trypsin in 50 mM ammonium bicarbonate overnight at 37 °C. Tryptic peptides were extracted from each gel with 50% acetonitrile, 5% acetic acid in water, dried and re-suspended in 20 μl of 2% acetonitrile, 0.1% formic acid in water for LC–MS/MS analysis.

### 2.4 LC–MS/MS analysis

Peptides from each gel fraction were separated on an Ultimate 3000 RSLC nano System utilising a PepMap C18 column, 2 µm particle size, 75 µm × 50 cm (Thermo Scientific, USA) and subsequently analysed on a QExactive mass spectrometer (Thermo Scientific, USA). Peptides were introduced into the mass spectrometer using an EASY-Spray™ nano source at a flow rate of 250 nl/min at approximately 400–600 bars. The 15 most intense precursors were selected for MS/MS analysis using higher-energy collisional dissociation (HCD) fragmentation and all fragmented precursor ions were actively excluded from repeated MS/MS analysis for 27 s.

### 2.5 Data analysis

#### 2.5.1 Protein identification and relative abundance

RNAseq data of the schizont stage of *T. parva* has recently been made available in GenBank (BioSample accession SAMN03981746) and used to improve the annotation of the genome (da Silva, pers. comm.). The format for open reading frame (ORF) locus tags in the re-annotated genome of the Muguga strain has been changed from TPXX_YYYY (Gardner et al., 2005) to TpMuguga_0Xg0YYYY, where X stands for the chromosome number and YYYY for the gene number. This study relied on the re-annotated genome and the new format for *T. parva* ORF locus tags has been adopted.

Raw MS/MS data files were analysed with Peaks software (Bioinformatics solutions) using a database containing all Uniprot database entries for *R. appendiculatus* and the re-annotated proteome of *T. parva* (a total of 16,969 protein entries with 4084 *T. parva* and 12,884 *R. appendiculatus* protein entries, July 2016). A peptide false discovery rate (FDR) of 1.2% was applied and only proteins with unique peptide identification were included in the final sporozoite proteome list.

In order to rank the identified proteins by their abundance in the sample, an Exponentially Modified Protein Abundance Index (emPAI) was calculated using Mascot (Matrix Science) (Ishihama et al., 2005). EmPAI is the exponential form of protein abundance index (PAI) minus 1 (emPAI = 10^PAI^ − 1) and is proportional to protein content in a protein mixture (Ishihama et al., 2005). PAI is calculated by dividing the number of observed peptides by the number of observable peptides per protein.

#### 2.5.2 Classification of subcellular localization of the identified *T. parva* sporozoite proteins

Proteins were classified according to their putative localization in the sporozoite using TargetP (Emanuelsson et al., 2000). Trans-membrane domains (TMDs) were predicted by TMHMM Server v. 2.0 (Krogh et al., 2001), signal peptides (SP) by SignalP 4.1 (Petersen et al., 2011) and glycosylphosphatidylinositol (GPI)-anchor signal by GPI-SOM (Fankhauser and Mäser, 2005).

#### 2.5.3 Classification of the identified *T. parva* sporozoite proteins by orthology

We clustered all sporozoite protein coding sequences of *T. parva* with the complete predicted proteome of *Plasmodium falciparum* into putative orthologous groups using the OrthoMCL standalone software Version 2.0.2 (Li et al., 2003). The blast step of OrthoMCL was performed using an E-value cut-off of 1e^−10^, and minimum 70% sequence identity over 75% sequence coverage. *Plasmodium falciparum* 3D7 release of 26-06-2017 sequence data was downloaded from PlasmoDB (Aurrecoechea et al., 2010)

## 3 Results

### 3.1 Sporozoite proteome determination and relative quantification of expressed sporozoite proteins

LC–MS/MS was used to identify proteins expressed in a sample of semi-purified *T. parva* sporozoites. A complete list of all the proteins identified in this study is provided in Supplementary Table S1. In total 4780 proteins were identified of which 2007 originated from *T. parva* and 2773 from *R. appendiculatus*. Most proteins (3847) were identified with two or more peptides, while 933 protein identifications were based on a single peptide match, which includes 296 *T. parva* proteins (Supplementary Table S2). Thus, there is higher confidence in the identification of 1711 expressed parasite proteins. Sequence coverage of over 85% was achieved for three proteins, glyceraldehyde 3-phosphate dehydrogenase NAD binding domain, histone H2B.1 and ribosomal protein S21e, all encoded by single copy genes. Protein TpMuguga_03g00168, annotated as a hypothetical protein, was identified by as many as 94 unique peptides.

The 2007 *T. parva* sporozoite proteins that were identified represent approximately 50% of the 4084 proteins predicted to be encoded by the re-annotated *T. parva* genome (da Silva, pers. comm). Of the 2007 proteins, 1287 were further ranked by calculation of relative abundance in the sample using emPAI values (Supplementary Table S3), a parameter that is useful for obtaining an overview of proteome profiles with a wide dynamic range of concentrations (30 fmol to 1.8 pmol/μl) (Ishihama et al., 2005). The 20 most abundant *T. parva* sporozoite proteins are shown in Table 1. Even within this data set, there is a large range in the abundance of parasite proteins with histones being the most abundant. Other housekeeping proteins, e.g., ribosomal proteins, heat shock protein 70, phosphoglycerate mutase and glyceraldehyde 3-phosphate dehydrogenase, feature in this list.

**Table 1:** Abundant *Theileria parva* sporozoite proteins. Proteins detected from whole cell lysates of sporozoites are ranked by relative abundance in the sample. Known antigens are in bold face.

ORF locus tag^a^	Annotation^b^	Mass (Da)	emPAI^c^
TpMuguga_04g00404	Histone H2B.1	12,040	7228.34
TpMuguga_04g00071	Histone H2B	13,682	900.39
TpMuguga_04g00675	Histone H4	11,362	457.35
TpMuguga_04g00690	Phosphoglycerate mutase 1 family	29,221	105.17
TpMuguga_04g00050	Ribosomal protein S19	17,150	99.38
TpMuguga_03g00655	Hypothetical protein	17,296	96.5
TpMuguga_04g00036	AhpC/TSA family	22,120	84.68
TpMuguga_02g00487	Ribosomal protein S6e	25,553	67.36
TpMuguga_02g00903	Actin	42,270	60.62
TpMuguga_04g00322	Histone H2A	13,498	60.26
TpMuguga_03g00067	Hypothetical protein	11,572	54.06
TpMuguga_01g00726	elF-Tu GTP binding domain	49,779	52.64
TpMuguga_01g00541	Hypothetical protein	24,796	52.36
TpMuguga_04g00383	GAPDH NAD binding domain	36,883	51.37
**TpMuguga_01g00701**	**RAP-1**	**70,441**	**47.73**
TpMuguga_02g00148	Heat shock 70 kDa protein	71,445	42.22
TpMuguga_04g00179	RanBP1 domain	38,982	41.34
TpMuguga_03g00700	Hypothetical protein	23,995	40.00
**TpMuguga_03g00287**	**Sporozoite P67 surface antigen**	**75,453**	**39.16**
TpMuguga_01g00067	Acyl CoA binding protein	10,043	39.11

a Open reading frame (ORF) locus tag refers to a unique *T. parva* gene identifier.

b Hypothetical protein is of unknown function; AhpC/TSA, alkyl hydroperoxide reductase subunit C/ thiol specific antioxidant family; elF, elongation factor; RAP-1, homolog of a *Theileria annulata* protein annotated as Rhoptry-associated protein 1; GAPDH, Glyceraldehyde 3-phosphate dehydrogenase; NAD, Nicotinamide adenine dinucleotide; RanBP1, Ran binding protein 1; CoA, co-enzyme A.

c emPAI, relative protein abundance in sample.

### 3.2 Identification of known *T. parva* antigens within the sporozoite proteome

The major circumsporozoite surface protein of *T. parva*, p67, which is a target of sporozoite neutralising antibodies (Dobbelaere et al., 1984), was identified among the 20 most abundant proteins (Table 1). Additional antigens that had been previously characterised were also identified in this study, e.g., p104 (Ebel et al., 1999), p150 microneme/rhoptry proteins (Skilton et al., 1998) and p32, a merozoite antigen (Skilton et al., 2000). We searched the sporozoite proteome data (Supplementary Table S1) for characterised schizont CD8 T cell antigens (Graham et al., 2006, Graham et al., 2008) and found evidence for expression of all known CTL antigens (reviewed in Nene and Morrison, 2016) in the sporozoite stage. In contrast to CD8 T cell antigens, CD4 T cell antigens still require better characterization. CD4 antigens are presented by intact schizont-infected cells (Brown et al., 1989) as well as cell extracts (Ballingall et al., 2000). Soluble cytosolic parasite antigens stimulatory to CD4 T cell clones with fractions ranging between 10 and 24 kDa (Brown et al., 1995) have been described, but further analysis and identification of the specific constituent proteins is required. However, among the most abundant sporozoite proteins we identified a protein that is homologous to a *Theileria annulata* protein annotated as rhoptry associated protein-1 (RAP-1) (Table 1). RAP-1 of *B. bigemina* has been shown to be a CD4 T cell antigen (Brown et al., 1999), suggesting that the *T. parva* RAP-1 is a potential CD4 T cell antigen.

### 3.3 *Theileria parva* sporozoite proteins associated with apicomplexan invasion of host cells

Sporozoite secretory organelle proteins are typically located in the micronemes, rhoptries and dense granules in *P. falciparum* (reviewed in Lindner et al., 2013). To determine whether these proteins are present in *T. parva* sporozoites, we clustered our identified *T. parva* sporozoite proteins, using OrthoMCL, into orthologous groups with all *P. falciparum* genes. A total of 1105 proteins of the 2007 *T. parva* proteins were defined as *P. falciparum* orthologs (Supplementary Table S4) and we searched this list for apical organelle proteins. We identified *T. parva* orthologs of eight rhoptry proteins, three microneme proteins and a dense granule protein (Table 2). Featured in the list are orthologs of *P. falciparum* apical membrane antigen 1 (AMA-1), a microneme protein essential during host cell invasion (Treeck et al., 2009), and cell-traversal protein for ookinetes and sporozoites (CelTOS), a malarial antigen mediating host cell invasion (Kariu et al., 2006). AMA-1 is a leading malaria vaccine candidate (Remarque et al., 2008), and orthologs of it are also found in *Neospora caninum* and *Toxoplasma gondii* (Zhang et al., 2007).

**Table 2:** Invasion organelle proteins. Proteins detected from whole cell lysates of *Theileria parva* salivary gland sporozoites are aligned by orthology with *Plasmodium falciparum* sporozoite proteins.

Orthologous group	Organism	ORF locus tag a	Gene name^b^	Annotation (reference)
*Rhoptry proteins*				
OG5_142870	*P. falciparum*	PF3D7_1452000	PfRON2	Rhoptry neck protein 2 (Cao et al., 2009)
	*T. parva*	TpMuguga_01g00014	–	Hypothetical protein
OG5_153587	*P. falciparum*	PF3D7_0817700	RON5	Conserved *Plasmodium* protein, unknown function (Curtidor et al., 2011)
	*T. parva*	TpMuguga_01g01161	–	Hypothetical protein
OG5_153563	*P. falciparum*	PF3D7_1347500	ALBA4	Conserved *Plasmodium* protein, unknown function (Sam-Yellowe et al., 2004)
	*T. parva*	TpMuguga_02g00645	–	Hypothetical protein
OG5_128020	*P. falciparum*	PF3D7_0932300	PfM18AAP	M18 aspartyl aminopeptidase (Lauterbach and Coetzer, 2008)
	*T. parva*	TpMuguga_01g01150	–	aspartyl aminopeptidase
OG5_145111	*P. falciparum*	PF3D7_0906000	RNaseII	RNB-like protein, putative (Sam-Yellowe et al., 2004)
	*T. parva*	TpMuguga_01g00396	–	Hypothetical protein
OG5_141731	*P. falciparum*	PF3D7_1361800	GAC	Conserved *Plasmodium* protein, unknown function (Sam-Yellowe et al., 2004)
	*T. parva*	TpMuguga_01g00092	–	Hypothetical protein
OG5_129273	*P. falciparum*	PF3D7_0814200	ALBA1	DNA/RNA-binding protein Alba 1(Lindner et al., 2013)
	*T. parva*	TpMuguga_03g00655		Hypothetical protein
OG5_154281	*P. falciparum*	PF3D7_1006200	ALBA3	DNA/RNA-binding protein Alba 3 (Lindner et al., 2013)
	*T. parva*	TpMuguga_03g00067		Hypothetical protein

*Microneme proteins*				
OG5_147452	*P. falciparum*	PF3D7_1133400	AMA1	Apical membrane antigen 1, AMA1 (Cowman and Crabb, 2006)
	*T. parva*	TpMuguga_01g00650	–	Apical membrane antigen 1
	*falciparum*			
OG5_171217	*P. falciparum*	PF3D7_1216600	CelTOS	Cell-traversal protein for ookinetes and sporozoites, putative (Kariu et al., 2006)
	*T. parva*	TpMuguga_01g00232	–	Hypothetical protein
OG5_135185	*P. falciparum*	PF3D7_0212900	–	Leu/Phe-tRNA protein transferase, putative (Lindner et al., 2013)
	*T. parva*	TpMuguga_02g00627	–	Leucyl/phenylalanyl-tRNA protein transferase

*Dense granules*				
OG5_126706	*P. falciparum*	PF3D7_0818200	–	14-3-3 protein, putative (Lindner et al., 2013)
	*T. parva*	TpMuguga_02g00607		Hypothetical protein

a Open reading frame (ORF) locus tag refers to a unique *T. parva* or *P. falciparum* gene identifier.

b Gene name is as defined in *P. falciparum*.

In *T. parva*, mobilisation of Ca^2+^ is necessary for successful sporozoite invasion of bovine lymphocytes and reagents that prevent Ca^2+^ mobilisation significantly curb zippering and internalisation stages of the entry process (Shaw, 1995). Table 3 shows 13 proteins identified in *T. parva* clustered with the *Plasmodium* proteins that play a role in calcium signalling, including calmodulin, an intracellular target for Ca^2+^ activation that acts on proteins such as guanylyl cyclases, protein kinases and phosphatases to aid in signal transduction.

**Table 3:** Proteins involved with calcium signalling. Proteins detected from whole cell lysates of *Theileria parva* salivary gland sporozoites are aligned by orthology with *Plasmodium falciparum* sporozoite proteins.

Orthologous Group	Organism	ORF locus tag^a^	Gene name^b^	Annotation (reference)
OG5_126800	*P. falciparum*	PF3D7_1434200	CAM	Calmodulin
	*T. parva*	TpMuguga_02g00717	–	Calmodulin
OG5_129380	*P. falciparum*	PF3D7_1027700	PfCEN1	Centrin-1 (Khan et al., 2005)
	*T. parva*	TpMuguga_01g00227	–	Centrin
OG5_152981	*P. falciparum*	PF3D7_1140000	CA	Carbonic anhydrase (Lindner et al., 2013)
	*T. parva*	TpMuguga_02g00412	–	Hypothetical protein
OG5_126600	*P. falciparum*	PF3D7_0717500	PfCDPK4	Calcium-dependent protein kinase 4 (Holder et al., 2012)
	*T. parva*	TpMuguga_01g01073	–	Calmodulin-domain protein kinase
	*P. falciparum*	PF3D7_1337800	CDPK5	Calcium-dependent protein kinase, (Dvorin et al., 2010)
	*T. parva*	TpMuguga_02g00399	–	Calcium-dependent protein kinase
OG5_131887	*P. falciparum*	PF3D7_1123100	CDPK7	Calcium-dependent protein kinase 7 (Lindner et al., 2013)
	*T. parva*	TpMuguga_04g00518	–	Protein tyrosine kinase
OG5_127599	*P. falciparum*	PF3D7_1436600	PKG	cGMP-dependent protein kinase (Lindner et al., 2013)
	*T. parva*	TpMuguga_03g00511		Protein tyrosine kinase
OG5_133188	*P. falciparum*	PF3D7_1138400	GCalpha	Guanylyl cyclase (Pradel, 2007)
	*T. parva*	TpMuguga_02g00848	–	Guanylyl cyclase
OG5_141757	*P. falciparum*	PF3D7_1246400	MTIP	Myosin A tail domain interacting protein (Jones et al., 2006)
	*T. parva*	TpMuguga_01g00513	–	Myosin light chain
OG5_126577	*P. falciparum*	PF3D7_1329100	MyoC	Myosin C(Tyagi et al., 2009)
	*T. parva*	TpMuguga_01g00774	–	Myosin C
OG5_126674	*P. falciparum*	PF3D7_1211900	ATPase4	Non-SERCA-type Ca^2+^ –transporting P-ATPase (Budu and Garcia, 2012)
	*P. falciparum*	PF3D7_0106300	ATP6	Calcium-transporting ATPase, putative (Budu and Garcia, 2012)
	*T. parva*	TpMuguga_01g00720	–	P-type ATPase
	*T. parva*	TpMuguga_02g00524	–	Calcium-transporting ATPase
OG5_127599	*P. falciparum*	PF3D7_0934800	PKAc	cAMP-dependent protein kinase catalytic subunit (Lindner et al., 2013)
	*T. parva*	TpMuguga_02g00378	–	cAMP-dependent protein kinase

a Open reading frame (ORF) locus tag refers to a unique *T. parva* or *P. falciparum* gene identifier.

b Gene name is as defined in *P. falciparum*.

### 3.4 Putative *T. parva* surface proteins within the sporozoite proteome

We used two methods to search for novel putative surface proteins. The first method was to use bioinformatics tools to predict the presence of GPI anchors and SP in proteins expressed by sporozoites. Only 12 proteins of the 2007 sporozoite proteins were found in this category (Table 4). The list includes TpMuguga_01g00939 (gp34), a protein that undergoes GPI modification when expressed in mammalian cells and a schizont surface protein (Xue et al., 2010). Since there is no direct evidence for addition of GPI anchors to parasite expressed proteins, we searched for enzymes of the GPI biosynthesis pathway encoded by the *T. parva* genome. Eight genes are essential for GPI synthesis in *P. falciparum* (Delorenzi et al., 2002). We found homologs of all eight genes in *T. parva* (TpMuguga_04g00759, TpMuguga_04g00881, TpMuguga_04g00525, TpMuguga_01g00169, TpMuguga_02g00201, TpMuguga_03g00397, TpMuguga_03g00846 and TpMuguga_02g00741) and identified that the latter four are expressed in the sporozoite proteome. This suggests the existence of GPI anchored proteins in *T. parva*, although this remains to be formally proven.

**Table 4:** Potential sporozoite surface proteins. Proteins detected from whole cell lysates of *Theileria parva* salivary gland sporozoites predicted to contain a glycosylphosphatidylinositol anchor and either transmembrane domain or signal peptide.

ORF locus tag^a^	Annotation^b^	Unique peptides^c^	emPAI^d^	TMD^e^	GPI-anchor^f^	SP^g^
TpMuguga_01g00438	Hypothetical protein	34	1.01	0	1	1
TpMuguga_01g00939	Hypothetical protein (gp34)	12	3.65	1	1	1
TpMuguga_01g00939	Hypothetical protein	12	3.65	1	1	1
TpMuguga_01g00972	Hypothetical protein	4	0.45	1	1	1
TpMuguga_01g01056	Merozoite antigen (p32)	12	6.78	0	1	1
TpMuguga_02g00412	Hypothetical protein	5	0.3	0	1	1
TpMuguga_02g00538	Hypothetical protein	31	1.34	1	1	1
TpMuguga_02g00553	Hypothetical protein	6	2.95	1	1	1
TpMuguga_02g00950	Hypothetical protein	2	<0.05	1	1	1
TpMuguga_03g00287	Sporozoite p67 surface antigen	53	39.16	1	1	1
TpMuguga_04g00437	104 kDa microneme/rhoptry antigen (p104)	60	12.35	1	1	1
TpMuguga_04g00615	Probable N-acetylglucosaminyl-phosphatidylinositol de-N-acetylase	2	<0.05	0	1	1

a Open reading frame (ORF) locus tag refers to a unique *T. parva* gene identifier.

b Hypothetical protein is of unknown function(s).

c Unique peptides refers to the total number of peptides identified matching the protein sequence that do not match any other protein in the database searched.

d emPAI, relative protein abundance in sample.

e Predicted using the TMHMM Server v. 2.0 (http://www.cbs.dtu.dk/services/TMHMM/).

f Predicted using the GPI-SOM (http://gpi.unibe.ch/).

g Predicted using the SignalP 4.1 server (http://www.cbs.dtu.dk/services/SignalP/).

Secondly, we compiled from the literature a list of surface-exposed *P. falciparum* proteins and determined if orthologs exist within the *T. parva* sporozoite proteome. We identified orthologs of AMA-1 (TpMuguga_01g00650) and a hexose transporter (TpMuguga_01g01069) which were both identified by specific biotinylation of *P. falciparum* and *Plasmodium yoelii* surface proteins (Lindner et al., 2013).

### 3.5 *Theileria parva* proteins as candidate manipulators of host cell signalling leading to transformation

Lymphocyte transformation characterised by cancer-like cell phenotypes is solely dependent on viable schizonts and requires the schizont proteins to interact with and manipulate pathways regulating lymphocyte apoptosis, proliferation and gene expression (Dobbelaere and Heussler, 1999, Tretina et al., 2015). We identified 17 proteins predicted to be manipulators of host cells (Shiels et al., 2006) in the sporozoite stage, suggesting that these proteins may be required immediately upon host cell entry. Transcripts for the genes encoding all 17 proteins are found in the RNAseq data from schizont-infected cells (see Section 2.5.1), although at different levels (Supplementary Table S5). Among the identified proteins are members of two multigene families, the Sub-telomere-encoded Variable Secreted Protein (SVSP) gene family and the *T. annulata* schizont AT-hook (TashAT) gene family, or *T. parva* host nuclear (TpHN) gene family. TashAT proteins display a high degree of sequence conservation in their DNA-binding domains (AT-hook) in *T. annulata* but not in *T. parva* and contain an N-terminal signal sequence for transport across the parasite plasma membrane (reviewed in Tretina et al., 2015). TashAT family proteins are implicated in regulation of host gene expression and as accessory factors for parasite transcription (Shiels et al., 2006). Other families featured are DEAD-box RNA helicases with members implicated in alteration of RNA secondary structures and promotion of proto-oncogene c-myc expression (e.g., DDX6) (Abdelhaleem, 2004), thioredoxin/glutaredoxin family that are redox regulators and influence important signalling pathways such as nuclear factor kappa light chain enhancer of activated B cells (NF-kB) and activator protein-1 (AP1) pathways that are both activated in *Theileria*-transformed cells (Shiels et al., 2006). In addition, we identified *T. parva* Schizont-derived Cytoskeleton-binding Protein (TpSCOP), a protein that activates host NF-κB and mitogen-activated protein kinase (MAPK) pathways, leading to resistance to programmed host cell death (Hayashida et al., 2010) and a peptidyl-prolyl isomerase, which has been demonstrated to interact with and lead to degradation of host ubiquitin ligase FBW7, thereby stabilising host c-JUN and promoting transformation of host cells (Marsolier et al., 2015). Interestingly, the bulk schizont RNAseq data identified a high level of anti-sense transcription of the TpSCOP gene (Supplementary Table S5), suggesting that expression of this gene in the schizont stage may be under differential control. Resolution of the significance of this observation will require analysis of cell-cycle synchronised samples.

### 3.6 Sporozoite proteins involved in glycolysis and tricarboxylic acid (TCA) cycles

**Fig. 1:**
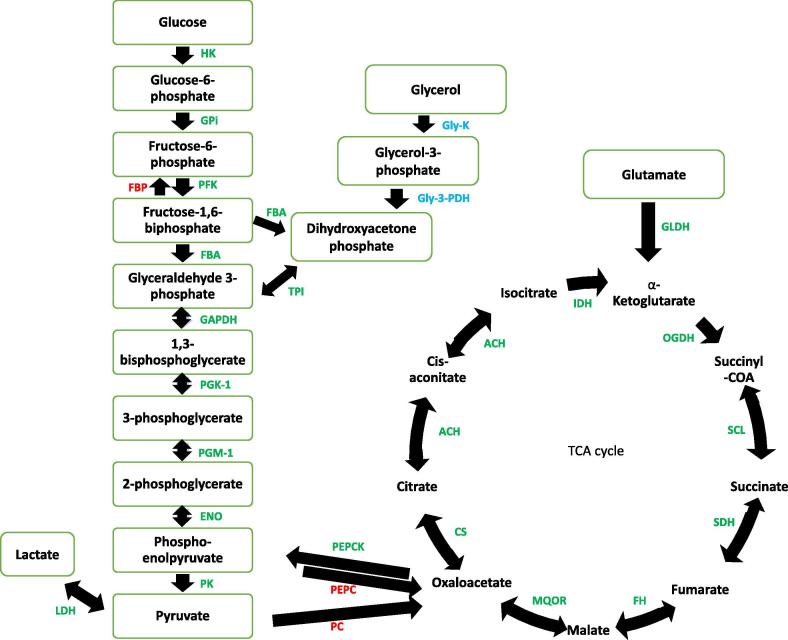
Most of *Theileria parva* proteins involved in glycolysis and tricarboxylic acid cycles were identified in this study. The glycolysis pathway is shown on the left and tricarboxylic acid cycle is shown on the right. Colour code for proteins: green, enzymes that were identified by MS; blue, enzymes encoded in the *T. parva* genome but not identified by MS; red, enzymes not encoded by the *T. parva* genome and not identified in this study. Enzymes identified by MS have open reading frame locus tags included in brackets (see below): HK, hexokinase (TpMuguga_01g00043); GPi, glucose 6-phosphate-isomerase (TpMuguga_03g00346); PFK, phosphofructokinase (TpMuguga_02g00577); FBP, fructose bisphosphatase; FBA, fructose bisphosphate aldolase (TpMuguga_01g00101); TPI, triosephosphate isomerase (TpMuguga_04g00464); Gly-3PDH, glycerol-3-phosphate dehydrogenase; Gly-K, glycerol kinase; GAPDH, glyceraldehyde phosphate dehydrogenase (TpMuguga_02g00858); PGK-1, phosphoglycerate kinase (TpMuguga_01g00965); PGM-1, phosphoglycerate mutase (TpMuguga_04g00690); ENO, enolase (TpMuguga_04g00700); PK, pyruvate kinase (TpMuguga_02g00134, TpMuguga_04g00607); LDH, lactate dehydrogenase (TpMuguga_01g01182); PC, pyruvate carboxylase; ACH, aconitate hydratase-1 (TpMuguga_01g01050); IDH, isocitrate dehydrogenase (TpMuguga_04g00620); OGDH, oxoglutarate dehydrogenase (TpMuguga_01g00262); SCL, succinyl coenzyme A ligase (TpMuguga_04g00660); SDH, succinate dehydrogenase (TpMuguga_01g00210); FH, fumarate hydratase-1 (TpMuguga_03g00078); MDH, malate dehydrogenase; MQOR, Malate: quinone oxidoreductase (TpMuguga_03g00758); CS, citrate synthase (TpMuguga_02g00666); PEPC, phosphoenolpyruvate carboxylase; PEPCK, phosphoenolpyruvate carboxykinase (TpMuguga_01g00495). (For interpretation of the references to colour in this figure legend, the reader is referred to the web version of this article.)

Proteins of the glycolytic pathway are conserved in most eukaryotic organisms and 10 genes encoding glycolytic enzymes have been found in *Theileria* genomes (Gardner et al., 2005, Pain et al., 2005). We identified expression of all the 10 enzymes of the glycolytic pathway in this study (Fig. 1) including lactate dehydrogenase (LDH), an enzyme that converts pyruvate, the last product of glycolysis, to lactate during low or no oxygen conditions. We also identified all the TCA cycle enzymes by MS (Fig. 1) with the exception that malate dehydrogenase is functionally replaced by malate-quinone oxidoreductase (Gardner et al., 2005), and was identified in the proteome data. Glutamate dehydrogenase was also identified in the proteome and glutamate has been suggested to be a supplementary intermediate for the TCA cycle (Gardner et al., 2005). The only functional link we have found between glycolysis and the TCA cycle is the presence of phosphoenolpyruvate carboxykinase. Neither glycerol kinase nor glycerol 3-phosphate dehydrogenase were identified, although *T. parva* encodes genes for both enzymes. These enzymes were identified with low levels in the schizont stage of *T. annulata* (Witschi et al., 2013).

### 3.7 Tick proteins

Searching the Uniprot database entries for *R. appendiculatus* with the LC–MS/MS data led to identification of 2773 tick proteins in addition to the *T. parva* proteins. The complete list of all the identified tick proteins is presented in Supplementary Table S1, but a detailed analysis of vector proteins will be reported elsewhere.

## 4 Discussion

In this study, we employed a DE-52 ion-exchange column purification method to semi-purify *T. parva* sporozoites from infected tick salivary glands. Although tick proteins constituted 58% (2773/4780) of the total number of proteins of all proteins identified by LC–MS/MS and the Peaks and Mascot software packages, we were able to identify 2007 *T. parva* proteins expressed in the sporozoite life-cycle stage. This represents ∼50% of the total predicted re-annotated *T. parva* proteome of 4084 proteins (see Section 2.5.1). Approximately 40% of these proteins are annotated as hypothetical proteins. Hence, our data provides evidence that real genes encode these proteins, although their functions remain unknown.

Studies on the proteome of *P. falciparum* sporozoite stages have reported identification of ∼13% (Lasonder et al., 2008), ∼19% (Florens et al., 2002) and ∼36% (Lindner et al., 2013) of the 5524 annotated *P. falciparum* genes using materials purified by DEAE-cellulose chromatography only (Florens et al., 2002, Lasonder et al., 2008), and on 17% w/v Accudenz cushion followed by DEAE-cellulose chromatography (Lindner et al., 2013). The range of vector contamination is an important component of such studies and varied depending on the method of parasite purification. Lasonder et al. (2008) reported 65–89% mosquito protein contamination in the samples analysed, while Lindner et al. (2013) reported 60.1% mosquito proteins in the sample, but had to perform double purification, initially on a 17% w/v Accudenz cushion then followed by DEAE-cellulose chromatography, to reduce mosquito protein contamination to 29% (Lasonder et al., 2008, Lindner et al., 2013). The presence of mosquito material in the sample was not discussed by Florens et al. (2002). We managed to achieve low levels of vector protein contamination with a single DE-52 column purification step.

Positive identification of proteins by MS confers confidence in their presence, especially when the proteins are identified by more than one unique peptide hit. Of the 2007 parasite proteins, 1708 were identified by more than one hit, providing a high degree of confidence in 85% of the data. For the purposes of our current analyses we have, however, reported on the entire data set of 2007 proteins. Failure to identify *T. parva* proteins does not directly imply their absence in the sample. Peptides from proteins with low abundance may fall below the detection limit or are simply not sequenced by the mass spectrometer due to limited dynamic range and/or speed of instrument acquisition. Furthermore, unknown, complex, or amino acid modifications leading to alteration of the peptide mass can only be detected in some cases by the Peaks software. Finally, trypsin digestion of proteins with low or irregular arginine/lysine content can lead to very short and very long tryptic peptides that fall out of the acquired mass range, and may therefore not be detected.

Identification of *T. parva* sporozoite proteins through MS has allowed us to assess the proteomic component of the mammalian-infective stage of the parasite. We have also calculated the emPAI using Mascot to estimate the relative abundance ranking of proteins in the sample, which revealed a very high range of abundance with histones ranked with an emPAI of 60–7200 for the different histone subunits to an emPAI index of 39 for the major circumsporozoite p67 protein. Subunits of two enzymes within the glycolytic pathway, phosphoglycerate mutase and glyceraldehyde 3-phosphate dehydrogenase, are among the 20 most abundant sporozoite proteins detected (Table 1). A high abundance of histones is not unusual. Lindner et al. (2013) reported histones as the third most abundant protein in *Plasmodium* sporozoite proteome after the circumsporozoite protein and mitochondrial ATP synthase, subunit beta (Lindner et al., 2013).

A number of known antigens were identified within the sporozoite proteome including p67, a leading vaccine antigen, and previously described components of secretory organelles (e.g., p104 and p150) (Table 4). RAP-1, a rhoptry antigen protective against *P. falciparum* infection in *Saimiri* monkeys (Ridley et al., 1990), was also identified (Table 1). Unexpectedly, the polymorphic immuno-dominant molecule (PIM), a well-known antigen (Toye et al., 1991), was not identified in this study even though p150, an antigen that is immunologically cross-reactive with PIM (Skilton et al., 1998), was identified. The PIM protein sequence contains a large repetitive domain rich in glutamine and proline amino acid residues that is devoid of lysine and arginine residues (Toye et al., 1991). An in silico digestion of PIM with trypsin leads to prediction of few detectable tryptic peptides (data not shown). This may explain why we did not detect it. All schizont proteins that have been identified as CTL antigens are also expressed in sporozoites (emPAI range of 0.76–31.69). Some of these antigens are encoded by housekeeping proteins (e.g., hsp90) and translation elongation initiation factor 1A, so expression by sporozoites of some CTL antigens is not unexpected. The finding that all the known CTL antigens are expressed by sporozoites raises the question of whether infection of dendritic cells by sporozoites results in direct presentation of antigen for priming CTL responses in vivo, provided the antigen load is sufficient.

A study on the proteome of *T. annulata* schizont stage, in which 798 proteins were identified, failed to identify any member of SVSP and TashAT families (Witschi et al., 2013). In contrast, we identified members of both families in the sporozoite stage (Supplementary Table S5). There is increasing evidence pointing to a role of these proteins in host-parasite interaction, for instance SVSP epitope-tagged TpMuguga_03g00882 protein expressed in mammalian cells was found to translocate into the host nucleus, pointing to potential role in transforming host cells (Schmuckli-Maurer et al., 2009). In addition, we identified other predicted host manipulation proteins such as DEAD-box RNA helicases, peptidases and two proteins, namely a peptidyl prolyl-isomerase and TpSCOP, demonstrated to play roles in host manipulation by stabilising c-JUN and activation of NF-kB, respectively (Shiels et al., 2006, Hayashida et al., 2010, Marsolier et al., 2015). Host cell manipulation is known to occur at the schizont stage and the finding of these proteins in the sporozoite stage suggests early expression of these proteins may be required, or multi-functionality of the proteins.

Sporozoite invasion of lymphocytes begins with binding events that trigger the mobilisation of Ca^2+^ within the sporozoites (Dobbelaere and Heussler, 1999). The free intracellular Ca^2+^ can be utilised by calmodulin to remodel itself structurally so as to act on downstream effectors such as calcium-dependent protein kinases (CDPKs), which have been shown to be useful for parasite infectivity. We detected both calmodulin and CDPKs in this study (Table 3). We also detected other calcium signalling proteins including guanylyl cyclases, cGMP-dependent protein kinases and cAMP-dependent protein kinases, all of which are important for downstream calcium signalling and have been shown to be essential for sporozoite invasion (reviewed in Dobbelaere and Heussler, 1999).

In motile apicomplexans such as *Plasmodium*, free calcium induces microneme secretion by sporozoites to aid in gliding motility and host invasion (Lindner et al., 2013). Although *T. parva* sporozoites are immotile (Shaw, 2003), we identified orthologs of *Plasmodium* microneme components, including AMA-1, CelTOS and Leu/Phe-tRNA protein transferase (Cowman and Crabb, 2006, Kariu et al., 2006, Lindner et al., 2013). These proteins may be involved solely in invasion processes, together with the detected rhoptry proteins (PfRON2, Conserved *Plasmodium* protein, PfM18AAP and RNB-like protein) and dense granules protein (14-3-3 protein) (Sam-Yellowe et al., 2004, Lauterbach and Coetzer, 2008, Cao et al., 2009, Curtidor et al., 2011, Lindner et al., 2013). Since most of these proteins are still classified as hypothetical in *T. parva*, they present an opportunity for further analysis in studies aiming to block parasite invasion of host cells.

In conclusion, these data establish the expression profile of *T. parva* sporozoite proteins, the only such profile available to date. One or more of the newly identified proteins, in particular putative surface proteins, may prove to be effective vaccine candidates and might be combined with the major surface protein p67 to induce broader anti-sporozoite immunity, and protection against ECF.
